# Paternal Occupational Exposure to 2,3,7,8-Tetrachlorodibenzo-*p*-dioxin and Birth Outcomes of Offspring: Birth Weight, Preterm Delivery, and Birth Defects

**DOI:** 10.1289/ehp.7051

**Published:** 2004-06-23

**Authors:** Christina C. Lawson, Teresa M. Schnorr, Elizabeth A. Whelan, James A. Deddens, David A. Dankovic, Laurie A. Piacitelli, Marie H. Sweeney, L. Barbara Connally

**Affiliations:** ^1^National Institute for Occupational Safety and Health, Cincinnati, Ohio, USA; ^2^Office of Global Health Affairs, Department of Health and Human Services, Hanoi, Vietnam

**Keywords:** birth defects, birth weight, congenital anomalies, dioxin, occupation, paternal exposure, preterm birth, TCDD

## Abstract

Agent Orange is a phenoxy herbicide that was contaminated with 2,3,7,8-tetrachlorodibenzo-*p*-dioxin (TCDD). We studied pregnancy outcomes among wives of male chemical workers who were highly exposed to chemicals contaminated with TCDD and among wives of nonexposed neighborhood referents. For exposed pregnancies, we estimated serum TCDD concentration at the time of conception using a pharmacokinetic model. The mean TCDD concentration for workers’ births was 254 pg/g lipid (range, 3–16,340 pg/g). The mean referent concentration of 6 pg/g was assigned to pregnancies fathered by workers before exposure. A total of 1,117 live singleton births of 217 referent wives and 176 worker wives were included. Only full-term births were included in the birth weight analysis (≥37 weeks of gestation). Mean birth weight among full-term babies was similar among referents’ babies (*n* = 604), preexposure workers’ babies (*n* = 221), and exposed workers’ babies (*n* = 292) (3,420, 3,347, and 3,442 g, respectively). Neither continuous nor categorical TCDD concentration had an effect on birth weight for term infants after adjustment for infant sex, mother’s education, parity, prenatal cigarette smoking, and gestation length. An analysis to estimate potential direct exposure of the wives during periods of workers’ exposure yielded a nonstatistically significant increase in infant birth weight of 130 g in the highest exposure group (TCDD concentration > 254 pg/g) compared with referents (*p* = 0.09). Mothers’ reports of preterm delivery showed a somewhat protective association with paternal TCDD (log) concentration (odds ratio = 0.8; 95% confidence interval, 0.6–1.1). We also include descriptive information on reported birth defects. Because the estimated TCDD concentrations in this population were much higher than in other studies, the results indicate that TCDD is unlikely to increase the risk of low birth weight or preterm delivery through a paternal mechanism.

Agent Orange is a phenoxy herbicide that was widely used as a defoliant in Vietnam. A mixture of the herbicides 2,4-D [(2,4-dichlorophenoxy)acetic acid] and 2,4,5-T [(2,4,5-trichlorophenoxy)acetic acid], Agent Orange was contaminated with 2,3,7,8-tetrachlorodibenzo-*p*-dioxin (TCDD). Most of the general population is exposed to low levels of TCDD, primarily through dietary intake of animal fats. Occupational exposure to TCDD occurred in the United States during the manufacture of Agent Orange, as well as among U.S. Vietnam veterans who were exposed to Agent Orange during Operation Ranch Hand. Because most of these workers and veterans were men, there has been heightened interest in male reproductive health outcomes associated with exposure to TCDD.

Extensive data on laboratory animals suggest that developing tissues are highly sensitive to TCDD, so associations between TCDD exposure and adverse reproductive outcomes are biologically plausible [Eskenazi and Kimmel 1995; National Academy of Sciences (NAS) 2003]. A recent review of TCDD by the NAS concluded that TCDD is one of the most toxic chemicals known to affect animals, although there is an extreme range of effects among species, and that the most sensitive time of exposure to TCDD is during fetal development (NAS 2003). There have been few studies of paternal TCDD exposure and birth weight or preterm delivery in humans, however. In the fourth biennial update of the health effects of Vietnam veterans of exposure to herbicides, therefore, the NAS concluded there was insufficient or inadequate evidence to determine whether there is an association between paternal herbicide exposure and low infant birth weight or preterm delivery (NAS 2003). The NAS further concluded that there was limited suggestive evidence of an association between paternal exposure to herbicides and spina bifida (NAS 1997), but the evidence was inadequate regarding other birth defects (NAS 2003).

Low birth weight has been associated with infant mortality as well as outcomes later in life such as asthma, lower IQ, and hypertension (Wilcox 2001). Low-birth-weight babies either are born preterm (< 37 weeks of gestation) or are full-term but small (Wilcox 2001). Historically, etiologic research on pregnancy outcomes, such as birth weight or birth defects, has focused on maternal and fetal exposures. However, paternal exposures could be related to adverse reproductive outcomes through genetic damage to the male germ cell, transfer of chemicals via seminal fluid, or exposure from chemicals that the father brings home from the workplace or hobbies (referred to as take-home exposure).

We studied the pregnancy outcomes among wives of male chemical workers who were highly exposed to chemicals contaminated with TCDD and among nonexposed neighborhood referents who participated in a cross-sectional medical study. Previous reproductive health analyses of this cohort reported subtle alterations in reproductive gonadotrophin and testosterone levels in male workers (Egeland et al. 1994) but no association between paternal TCDD exposure and spontaneous abortion or sex ratio (Schnorr et al. 2001). In the present study we evaluated the association between paternal exposure to TCDD at the time of conception and birth weight and preterm delivery of offspring. We also describe birth defects as reported in maternal interviews.

## Materials and Methods

### Study population.

The reproductive health study was conducted as part of a cross-sectional medical study, described previously in detail (Sweeney et al. 1989, 1993). Briefly, the study was conducted in 1987–1988 by the National Institute for Occupational Safety and Health (NIOSH) and examined workers from plants in New Jersey and Missouri. Workers were exposed to TCDD during the production of sodium trichlorophenol or one of its derivatives, such as hexachlorophene [2,2′-methylene-bis-(3,4,6-trichlorophenol)] or 2,4,5-T, which was used to formulate Agent Orange.

For comparison, referents with no self-reported occupational exposure to TCDD were selected from the workers’ neighborhoods, matched on age (± 5 years), race, and sex. Selection of worker-matched referents was based on a procedure requiring neighborhood door-to-door solicitation by trained interviewers. Each eligible resident was assigned a random number from 1 to 6 (*n* = 938). Selection was based on the matching referent’s numerical position in the random sequence and continued until the sample size was met (Sweeney et al. 1989).

A questionnaire was used to collect information on health status and risk factors. Subjects were also asked to participate in a medical examination, which included drawing blood for determination of serum TCDD. The methods of serum collection, analytical methods, and quality control standards have been published previously (Fingerhut et al. 1989, 1991; Patterson et al. 1986, 1989; Turner et al. 1989). The study was voluntary, and informed consent was obtained from all study subjects.

Current and former wives/partners (hereafter referred to as wives) of male participants were contacted and administered a telephone interview, which collected detailed information on reproductive history, medical history, lifestyle factors, and occupational factors. Data for 14 women who worked at the plants and their referents were not included in the analysis because of the small number of births.

### Exposure assessment.

Dates of employment in TCDD-related processes defined the exposure period (Piacitelli et al. 1992). Pregnancies conceived after the father’s first date of exposure were considered exposed, whereas referent pregnancies and pregnancies conceived before the fathers’ exposure at the plants (hereafter referred to as preexposure pregnancies) were considered unexposed.

For exposed pregnancies, we estimated the worker’s serum TCDD concentration at the time of conception using a pharmacokinetic model (Dankovic et al. 1995; Thomaseth and Salvan 1998). This model was based on the following factors: serum TCDD concentration at the time of examination, dates of employment in TCDD-related processes, body mass index (BMI) measured by NIOSH at the time of examination, and BMI measured by the employer during employment. Analyses for lipid-adjusted TCDD concentrations were calculated on a lipid weight basis because TCDD accumulates in the lipid stores of the body (Fingerhut et al. 1989). BMI change over time was modeled as a continuous function of age (Thomaseth and Salvan 1998). Data for workers who had both BMI values were used to create a linear regression model of BMI change over time using age at first employment, age at examination, and BMI at examination. This model was then applied to workers who had only one BMI value (*n* = 23).

The pharmacokinetic modeling technique has the advantage that it allowed for changes in individual body burden over time. The model assumed that there was no occupational exposure to TCDD after termination of employment, with a continuation of the background exposure to TCDD throughout life. In general, the modeled concentrations of TCDD in workers increased from first exposure to last exposure and then gradually declined to the concentration measured at the examination (Schnorr et al. 2001).

TCDD serum measurements were obtained for a random sample of 79 referents at examination. Because the referent serum concentrations were assumed to be the accumulation of a lifetime of background environmental exposures, we assigned the TCDD serum values from the examination to each referent pregnancy. For the remaining referents, the median referent value of 6 pg/g lipid was assigned (Piacitelli et al. 1992; Thomaseth and Salvan 1998). Pregnancies fathered by workers before exposure were also assumed to have exposure at the background level and so were assigned the median referent value of 6 pg/g.

### Outcome definitions.

We analyzed three outcome variables for this study: birth weight, preterm delivery, and birth defects. Only live singleton births were included in the birth weight and preterm delivery analyses. Furthermore, the birth weight analysis was conducted only among full-term births. Although previous studies of birth weight and environmental or occupational exposure have included preterm births, the most current literature recommends analyzing birth weight only among full-term births for two reasons: An exposure that affects fetal growth does not necessarily affect preterm delivery, and among term births, the influence of gestational age is minor (Wilcox 2001). For the birth defects analysis, only singleton births and stillbirths were included. Pregnancies not fathered by the study males were excluded in all analyses.

We requested birth certificates for all births, as well as neonatal death certificates and medical records where applicable. Birth certificates were obtained for 82% of the births (86% for worker births and 77% for referent births). Birth weight as recorded on the birth certificates was used when available. If no certificate was available, then the mother’s report of her child’s birth weight was used. Among women who had both birth certificates and mother’s report, the correlation coefficient was 0.91. We analyzed birth weight in grams to be consistent with previous literature.

We defined full-term birth as a live birth of ≥37 completed weeks from last menstrual period (LMP) or no more than 3 weeks before due date. For gestational age, we used the mother’s response to the question “Was the baby born on time, early, or late,” and if early or late, “by how many weeks?” We compared the mother’s report of gestational age to what was reported on the birth certificate, when available, and found that 49% were in agreement within the exact week and an additional 25% agreed within 1 week, leaving 26% who disagreed by > 1 week. We found little difference among exposure groups when comparing the percentages of those who disagreed by > 1 week (referents, 25%; pre-exposure workers, 27%; postexposure worker pregnancies, 28%).

We used the criteria described by Erickson et al. (1984) to define infants with major birth defects—that is, those defects that would affect survival, require substantial medical care, or result in marked physical or psychological challenges.

### Statistical analysis.

For the birth weight analysis, we modeled the primary independent variable, TCDD concentration, both as a continuous variable using the log and as a categorical variable (referents, < 20 pg/g, 20 to < 255 pg/g, ≥255 pg/g) using dummy variables. Unlogged TCDD and log-transformed TCDD gave similar results, but the model fit better with logged data, indicating a transformation was necessary. Therefore, the log-transformed TCDD data are presented to linearize the regression model. We selected the < 20-pg/g category as the lowest TCDD category because all of the referent serum samples were < 20 pg/g. The other categories were created based on the distribution of the TCDD estimates in our previous report (Schnorr et al. 2001). The pregnancies fathered by the workers fell into the four exposure categories as follows: 20% of pregnancies had serum values < 20 pg/g; 20% of pregnancies had serum values ≥1,120 pg/g; and the remaining 60% of the pregnancies were split equally into two categories, 20 to < 255 and 255 to < 1,120. For the analyses in this report, the upper two categories are combined into > 255 pg/g.

Repeated-measures analyses of variance were performed for the birth weight analysis using the SAS PROC MIXED procedure (SAS Institute, Inc., Cary, NC, USA) to account for the lack of independence among multiple pregnancies per mother. Restricted maximum likelihood was used to estimate the parameters, and a compound symmetric variance covariance structure was assumed.

To analyze preterm birth, we dichotomized gestational age into two categories: < 37 weeks or ≥37 weeks. The primary independent variable, TCDD, was modeled as a continuous (log) variable (too few subjects in the preterm category prevented analysis by TCDD categories). Repeated-measures analyses of variance were performed using the SAS PROC GEN-MOD procedure to account for the lack of independence among multiple pregnancies per mother.

For the birth weight and preterm analyses, we used univariate analyses to search for medical, lifestyle, and exposure factors that could potentially confound multivariate analyses. Potential confounders included mother’s medical conditions, prenatal medication use, alcohol consumption, cigarette smoking, maternal age at conception, parity, year of the birth, and prenatal weight gain; length of gestation; infant sex; accident, injury, or falls during pregnancy; maternal workplace factors such as occupational exposure to chemicals, prolonged standing, and heavy lifting; and mother’s and father’s education, race, and ethnicity. A variable was considered a potential confounder and retained for further modeling if it was significantly related to both log TCDD concentration and the outcome (*p* < 0.20) or changed the TCDD estimate by more than 15%. The variables that were retained for multivariate modeling for the birth weight analysis were sex of the infant, education of the mother, parity, cigarette smoking during pregnancy, and length of gestation. Inclusion in the model of the squared term for gestational length did not improve the model. The variables retained for the preterm birth analysis included having an accident, injury, or fall during pregnancy; mother’s age; and cigarette smoking and medication use during pregnancy.

For the birth defects analysis, we reviewed 1,153 live births and 13 stillbirths for a total of 1,166 births. We attempted to confirm all birth defects with medical records and/or vital or death records; however, because not all birth defects were confirmed, and because the maternal recall period could be several years, we only report those that were considered to be serious enough have a high likelihood of accurate recall. Small numbers of birth defects (*n* = 41) limited a meaningful analysis of birth defect categories by TCDD concentration, so only descriptive data of birth defects are reported.

## Results

Characteristics of the study participants have been reported in previous publications (Schnorr et al. 2001; Sweeney et al. 1989, 1993). A total of 281 workers (70% of the 400 living locatable workers) participated in the medical examination. Nine hundred thirty-eight men from matching neighborhoods were eligible to participate. Random selections of the eligible referents were invited to participate until 260 were enrolled. More than half (62%) of the individuals who were first or second in the random sequences of 1–6 agreed to participate in the study (Sweeney et al. 1989). Among living current and former wives of these men, we interviewed 245 (77.5%) of the workers’ wives and 221 (73.9%) of the referents’ wives.

Of those who were interviewed, 176 of the worker wives and 217 of the referent wives had at least one singleton live birth and were included in the birth weight analyses. Most of the study population was Caucasian race (89.4% referent wives and 90.3% worker wives), and a small percentage were of Hispanic ethnicity (1.8% of referent wives and 3.1% of worker wives). A higher percentage of the referent wives had more than a high school education (38.2 vs. 34.5% of the worker wives).

Included in the birth weight analyses were a total of 1,117 live full-term births, 604 to referent wives and 513 to worker wives. Of the babies fathered by workers, 221 were conceived before the father was exposed to TCDD at the study company (preexposure births), and 292 were conceived during or after exposure and are considered exposed births. Table 1 shows demographic characteristics of referent births and preexposure and exposed births. Mothers of preexposure births were younger than mothers of referent or exposed births (*p* < 0.01). Births conceived during or after fathers’ exposure to TCDD had a higher average exposure to cigarette smoke compared with referents (*p* = 0.04), although the percentages of mothers who smoked were similar. Alcohol use was higher in exposed pregnancies compared with referent and preexposure births, although the differences were not statistically significant.

The median estimated TCDD concentration for exposed births was 254 pg/g (range, 3–16,340 pg/g; Table 1). The median TCDD serum concentration for referent fathers who participated in the medical exam was 6 pg/g (range, 2–19), which was the value assigned to all referent and preexposure births. As reported previously (Schnorr et al. 2001), there was a lower percentage of male births in the pre-exposure group (50.7%) than in the referent (54.5%) and exposed (56.2%) groups, although these differences were not statistically significant.

The same percentage of referent and worker wives worked during their pregnancies (28%), and few reported exposure to chemicals or radiation during the pregnancies (0.3–3.0%; data not shown). A portion of the wives worked in jobs that involved physical stressors such as heavy lifting, continuous standing, or use of vibrating tools (12% referent births, 10% pre-exposure births, and 8% exposed births).

**Figure 1:**
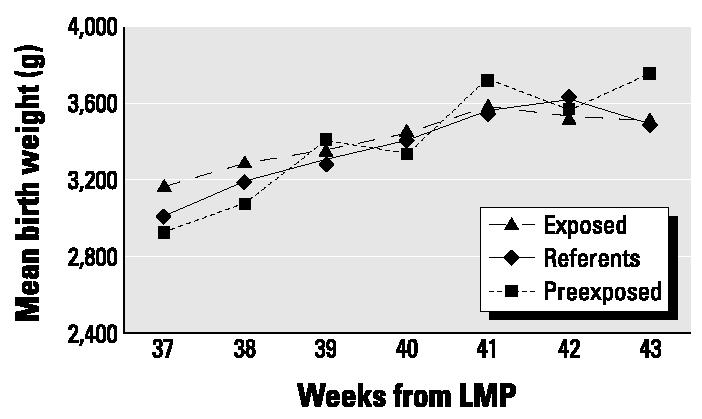
Mean birth weight versus weeks of gestation by exposure category.

Mean birth weights were similar in the three exposure groups (mean ± SD: referent births, 3,420 ± 490 g; preexposure births, 3,347 ± 485 g; exposed births, 3,442 ± 507 g). Figure 1 shows average birth weight by length of gestation for referent births and preexposure and exposed worker births. The graph is limited to gestational length of 37–43 weeks because of small numbers in the other weeks, as shown in Table 2. The three exposure groups presented in Figure 1 show similar mean birth weight patterns.

The results of a crude analysis show a small but statistically significant birth weight increase of 25 g with each increase in the log TCDD concentration (*p* = 0.01). Thus, for every 2- to 3-fold increase in TCDD, there was a 25-g increase in birth weight. There was no effect of continuous (data not shown) or categorical TCDD concentration (Table 3) on birth weight when adjusting for the following confounding variables: sex of the infant, education of the mother, parity, cigarette smoking during pregnancy, and length of gestation.

During the time of their husband’s employment, some of the workers’ wives may have been exposed directly to TCDD via contaminated clothing or vehicles (take-home exposure). To examine this, we restricted the analysis to the subset of births whose gestations overlapped the father’s dates of exposure at the plant. For the referents in this subanalysis, we included only births in which at least 1 day of the pregnancy occurred during the dates the two plants were in operation. The crude analysis showed a small increase in birth weight of 22 g (SE = 14) with each increase in the log of TCDD (*p* = 0.1). The adjusted analysis using TCDD as a continuous variable, however, showed a statistically significant increase of 31 g (SE = 14) in birth weight with each increase in the log of TCDD concentration(*p* = 0.03). In the categorical analysis (Table 4), the highest category of paternal TCDD (≥255 pg/g) showed an increase of 130 g (SE = 76) compared with the referents (*p* = 0.09). When we further restricted the analysis to pregnancies in which the entire pregnancy occurred during the father’s occupational exposure, or dates of plant operation for the referents, the results were similar to those shown in Table 4.

Because there were some differences among referent mothers, mothers of preexposed births, and mothers of exposed births in variables such as maternal age and parity (Table 1), we conducted an analysis of birth weight using the final adjusted model but limited only to exposed births (i.e., pregnancies conceived by workers during or after exposure; *n* = 287). This analysis showed a statistically significant increase in birth weight of 38 g with each increase in the log of TCDD concentration (*p* = 0.04).

There were 59 worker wives who had births in both exposure periods (preexposure and during/after exposure), with a total of 219 births (110 conceived preexposure and 109 conceived during or after exposure). An analysis of this subset showed that the mean birth weight for preexposure births was lower (mean ± SD, 3,388 ± 444 g) compared with exposed births (3,524 ± 563 g), similar to the main analysis. As would be expected, mean parity was also different between preexposure and exposed pregnancies (0.9 and 2.6, respectively). The difference in birth weight between the two exposure periods was not statistically significant after adjusting for parity. There was no difference in length of gestation or sex ratio between the two exposure periods in this subset.

To examine preterm births, we included all live births in the cohort (*n* = 1,153), consisting of 618 referents births, 238 preexposure births, and 297 exposed births. Of the 51 preterm births in the cohort (4.4%), 22 were among the 618 referent births (3.6%), 21 were among the 238 preexposure births (8.8%), and 8 were among the 297 exposed births (2.7%). A crude analysis of the preterm births showed a somewhat protective association with TCDD (log) concentration at LMP [odds ratio (OR) = 0.8; 95% confidence interval (CI), 0.6–1.0]. Results changed little when adjusted for accident during pregnancy, age of the mother, cigarette smoking during pregnancy, and medication use during pregnancy (OR = 0.8; 95% CI, 0.6–1.1; results not shown).

Table 5 shows selected birth defects that were reported on the maternal interviews, stratified by whether or not the father was exposed. The birth defect categories include central nervous system (CNS) defects, cardiovascular defects, genitourinary defects, clubfoot, hip and lower limb defects, orofacial clefts, and Down syndrome.

Six major CNS defects were reported: one case of spina bifida, two anencephalus cases, two hydrocephalus cases, and one case of multiple congenital anomalies. Two CNS cases were referents, two were in the background category (< 20 pg/g), one was in the low exposure category (20 to < 255 pg/g), and one case (spina bifida) was in the highest exposure category (≥1,120 pg/g). Four of these cases were confirmed. Two cases were not confirmed, specifically the spina bifida case and one of the hydrocephalus cases, because the records were not available. The mother of the spina bifida case reported that the child was diagnosed at age 5, with “neurologic impairment, spina bifida, and cerebral palsy.”

There were three cardiovascular defects, including tetralogy of Fallot, single ventricle, and Wolf-Parkinson-White syndrome. All three cases were in the referent or background (< 20 pg/g) categories.

## Discussion

The results of our analyses do not support a causal relationship between low birth weight and high paternal TCDD exposure. In fact, subanalyses showed a positive association between paternal dioxin exposure and the birth weight of offspring. There are few previous studies of paternal exposure to TCDD and birth weight. Michalek et al. (1998) studied Vietnam veterans who were exposed to Agent Orange and TCDD during Operation Ranch Hand. The median estimated TCDD concentration at the time of conception among Ranch Hand veterans was 79 ppt, with a range from 0 to 1,425 ppt (Michalek et al. 1998). The risk of intrauterine growth retardation was not increased in any Ranch Hand exposure category. In a study of sawmill industry workers, no increase was found in risk for lower birth weight in wives of men occupationally exposed to dioxin-contaminated chlorophenols, as measured by expert raters’ estimations of hours of exposure (Dimich-Ward et al. 1996). Although the chlorophenols in that study were not contaminated with TCDD, the workers were exposed to other polychlorinated dioxins. A study of Australian veterans who served in Vietnam showed a higher prevalence of low birth weight (< 2,500 g) among veterans than among controls (5.8% and 3.7%, respectively), although the difference was not statistically significant (Field and Kerr 1988). Multivariate analyses were not presented in that study, and no data on exposure were collected. Studies of maternal TCDD exposure and birth weight have had contradictory results (Eskenazi et al. 2003; Kimbrough and Krouskas 2001).

Overall, the proportion of preterm births in the present population was low (4.4%), and it was lower among exposed births (2.7%) than among preexposure births (8.8%) or referent births (3.6%). Multivariate analysis showed a somewhat protective effect of paternal TCDD concentration with respect to preterm birth. In a previous study of the Ranch Hand veterans, there was a moderate increase in risk of preterm birth among children in the high and background (unexposed) categories compared with referents, although there was no increased risk in the low exposure category, suggesting that the risk was not likely due to TCDD exposure (Michalek et al. 1998).

Our data were too limited to present statistical analysis of reported birth defects and paternal TCDD exposure, although descriptive information on birth defects did not suggest any relationship to TCDD. The NAS concluded in 1996 that there was limited suggestive evidence of an association between paternal exposure to herbicides and spina bifida, although the evidence was inadequate regarding other birth defects (NAS 1997). The NAS conclusion was based on three high-quality studies of Vietnam veterans: the Centers for Disease Control and Prevention (CDC) Birth Defects Study (Erickson et al. 1984), the CDC Vietnam Experience Study (CDC 1989), and the Ranch Hand study (Wolfe et al. 1995). All three studies suggested an association between herbicide exposure and an increased risk of spina bifida in offspring. In our study, there was one reported case of spina bifida. The medical records were not available to confirm the case, and the mother reported that it was not diagnosed until age 5; however, it is interesting to note that the father’s exposure was in the highest category of TCDD.

Many previous occupational studies of paternal exposure to dioxin and birth defects suffer from small numbers and inexact exposure methods. Two studies did not find any association between birth defects and occupational exposure to TCDD or 2,4,5-T, but both acknowledged limited statistical power (Smith et al. 1982; Townsend et al. 1982). In a study of sawmill workers, paternal exposure to dioxin-contaminated chlorophenol was associated with increased risks for congenital anomalies of the eye, anencephaly, spina bifida, and genital defects (Dimich-Ward et al. 1996).

Our study’s limitations include a lengthy recall period and the reliance on maternal report of gestational age, birth defects, and birth weight when birth certificates or medical records were not available. However, previous literature shows that mothers’ recall of birth weight is generally found to be accurate (Sanderson et al. 1998; Tomeo et al. 1999; Troy et al. 1996). Correlation coefficients between mother’s report of birth weight and birth weight recorded on the birth certificate ranged from 0.84 to 0.89, even when recall was two to four decades after birth (Sanderson et al. 1998; Troy et al. 1996) with a mean difference in birth weight of −25 g (Tomeo et al. 1999). When we conducted a subanalysis that excluded those individuals (18%) for whom birth weight was not confirmed by certificate, the results were similar. We conducted another subanalysis of birth weight in which the definition of full term was estimated from birth certificates rather than mothers’ questionnaires (excluding 250 additional births) that also showed similar results. Another limitation of our study is that serum TCDD was measured several years after the pregnancies, and therefore, the concentrations at LMP are estimates. However, we used a pharmacokinetic model to estimate concentrations at LMP, allowing for changes in individual body burden over time.

Because of the random sequencing procedure used in this study, we could not calculate a true participation rate for male referents. To address participation bias, a telephone interview was attempted with all of the male workers who refused to be examined and a 10% random sample of the referents who refused to participate. Of the 115 refusant workers and 129 refusant referents who were contacted, 68 (57%) and 99 (77%), respectively, agreed to be interviewed. The differences in reporting of selected chronic diseases between participants and nonparticipants were not statistically significant (Calvert et al. 1991, 1992, 1999). No significant difference in age was found between participants and refusants (Calvert et al. 1999). In addition, because male subjects agreed to participate in a cross-sectional medical health study and then later were asked to enroll in the reproductive health study, we do not anticipate that male refusants would differ from participants with respect to reproductive outcomes.

The men in our occupational study group were exposed to TCDD at substantially higher concentrations than other cohorts, with estimated concentrations at the time of conception ranging up to 16,340 pg/g. The strengths of our study include biologic measurements of internal dose and a pharmacokinetic modeling technique. Another strength is that we were able to adjust for confounding variables that were collected with a telephone interview. In addition, most of our outcome data were verified by birth certificate and/or medical records.

In conclusion, these results do not support a relationship between paternal TCDD exposure and lowered birth weight or preterm delivery. Because the estimated TCDD concentrations in this population were much higher than in other studies, the results indicate that TCDD is unlikely to increase the risk of low birth weight or preterm delivery through a paternal mechanism.

## Figures and Tables

**Table 1:** Selected characteristics of live full-term*^a^* births.

		Workers	
Variable	Referents (*n* = 604)	Preexposure (*n* = 221)	During exposure (*n* = 292)
Age of mother			
Mean ± SD	26.1 ± 5.4	23.4 ± 4.6	27.4 ± 5.3
Range	13.2–43.7	15.3–38.0	16.1–43.1
Year of conception			
Mean	1959	1956	1965
Range	1935–1987	1935–1971	1951–1987
Mother smoked cigarettes during pregnancy			
No. (%)	142 (23.7)	59 (26.9)	90 (31.0)
Cigarettes, average number per week			
Mean ± SD	23.2 ± 52.1	32.0 ± 63.9	37.5 ± 64.5
Range	0–280	0–280	0–280
Mother drank alcohol during pregnancy			
No. (%) pregnancies	182 (31.6)	44 (19.9)	112 (38.4)
Drinks per month			
Mean	2.2	1.2	3.6
Range	0–60	0–120	0–180
Paternal TCDD pg/g at conception			
Median	6	6*^b^*	254
Range	2–19		3–16,340
Parity			
Mean ± SD	1.5 ± 1.6	1.0 ± 1.2	1.8 ± 1.5
Range	0–9	0–6	0–8
Sex of infant: males			
No. (%)	329 (54.5)	112 (50.7)	164 (56.2)

a Gestational length ≥37 weeks.

b All preexposure births were assigned the median referent value of 6 pg/g.

**Table 2:** Number of births per length of gestation by exposure*^a^* category.

	Length of gestation (weeks)									
Exposure category	≤36	37	38	39	40	41	42	43	44–45	Total
Referents	22	20	35	40	368	41	55	19	18	618
Preexposed	21	15	12	7	142	14	20	6	1	238
Exposed	8	11	16	17	171	31	27	9	7	297

a Preexposure infants were conceived before the father worked in a dioxin-exposed job. Exposed infants were conceived during or after the father worked in a dioxin-exposed job.

**Table 3:** Mean difference in birth weight among term*^a^* infants by paternal TCDD exposure category.

	Mean difference in birth weight compared with referents (g)			
	Crude analysis		Adjusted analysis*^b^*	
TCDD category	No.	Difference	No.	Difference
Referent pregnancies (mean ± SE)	596	3,401 ± 27	592	3,402 ± 26
< 20 pg/g*^c^* (mean difference ± SE)	264	−57 ± 46	262	−8 ± 44
20 to 254 pg/g (mean difference ± SE)	98	−59 ± 62	98	−42 ± 59
≥255 pg/g (mean difference ± SE)	144	120 ± 55*	142	83 ± 52

a Gestational age ≥37 weeks.

b Adjusted for sex of the infant, education of the mother, parity, cigarette smoking during pregnancy, and length of gestation.

c This category includes preexposed and exposed births.

* *p*-Value ≤0.05 compared with referents.

**Table 4:** Mean difference in birth weight among term infants by paternal TCDD exposure category during employment.*^a^*

	Mean difference in birth weight compared with referents (g)			
	Crude analysis		Adjusted analysis*^b^*	
TCDD category	No.	Difference	No.	Difference
Referent pregnancies (mean ± SE)	334	3,397 ± 33	330	3,393 ± 32
< 20 pg/g (mean difference ± SE)	27	−182 ± 93*	26	−146 ± 91
20 to 254 pg/g (mean difference ± SE)	20	73 ± 105	20	156 ± 101
≥255 pg/g (mean difference ± SE)	51	74 ± 80	50	130 ± 76

a Pregnancies where at least 1 day in the pregnancy occurred during the father’s employment. For referents, pregnancies occurring during the dates the plants were manufacturing chemicals contaminated with 2,3,7,8 TCDD.

b Adjusted for sex of the infant, education of the mother, parity, cigarette smoking during pregnancy, and length of gestation.

* *p*-Value ≤0.05 compared with referents.

**Table 5:** Number of selected birth defects reported among 1,166 live born and stillborn infants.

Type of defect	Referents and preexposed*^a^*	Exposed*^a^*	Total reported	Total confirmed*^b^*
CNS	2	4	6	4
Cardiovascular	1	2	3	3
Genitourinary^c,d^	5	3	8	5
Clubfoot*^d^*	9	3	12	3
Unspecified hip and lower limb	5	1	6	2
Cleft lip and/or palate	2	1	3	1
Down’s syndrome	3	0	3	2
Total selected reported birth defects	27	14	41	20

a Preexposed infants were conceived before the father worked in a dioxin-exposed job. Exposed infants were conceived during or after the father worked in a dioxin-exposed job.

b Confirmation from vital or death records or medical records.

c Genitourinary defects included anorchism (*n* = 2), hypospadias (*n* = 3), adrenogenital syndrome (*n* = 1), a kidney defect (*n* = 1), and ureteropelvis obstruction (*n* = 1).

d One infant had clubfoot and hypospadias.
